# The Critical Role of Genome Maintenance Proteins in Immune Evasion During Gammaherpesvirus Latency

**DOI:** 10.3389/fmicb.2018.03315

**Published:** 2019-01-09

**Authors:** Océane Sorel, Benjamin G. Dewals

**Affiliations:** ^1^Immunology-Vaccinology, Department of Infectious and Parasitic Diseases, Faculty of Veterinary Medicine-FARAH, University of Liège, Liège, Belgium; ^2^Department of Molecular Biology and Biochemistry, University of California, Irvine, Irvine, CA, United States

**Keywords:** herpesvirus, viral latency, genome maintenance protein, immune evasion, antigen presentation, viral proteins

## Abstract

Gammaherpesviruses are important pathogens that establish latent infection in their natural host for lifelong persistence. During latency, the viral genome persists in the nucleus of infected cells as a circular episomal element while the viral gene expression program is restricted to non-coding RNAs and a few latency proteins. Among these, the genome maintenance protein (GMP) is part of the small subset of genes expressed in latently infected cells. Despite sharing little peptidic sequence similarity, gammaherpesvirus GMPs have conserved functions playing essential roles in latent infection. Among these functions, GMPs have acquired an intriguing capacity to evade the cytotoxic T cell response through self-limitation of MHC class I-restricted antigen presentation, further ensuring virus persistence in the infected host. In this review, we provide an updated overview of the main functions of gammaherpesvirus GMPs during latency with an emphasis on their immune evasion properties.

## Introduction

Herpesviruses are enveloped double-stranded DNA viruses that are in general responsible for persistent infections in a large number of animal species. In 2008, the International Committee on Taxonomy of Viruses (ICTV) created the order *Herpesvirales* comprising three families: the family *Malacoherpesviridae* composed of viruses infecting molluscs such as oysters, the family *Alloherpesviridae* composed of viruses infecting fish species and amphibians, and the predominantly studied family *Herpesviridae* that includes viruses of mammals and birds, itself classified into the three subfamilies *Alpha*-, *Beta*-, and *Gammaherpesvirinae*. A hallmark of all herpesviruses is their unique capacity to induce lifelong infection through establishing and maintaining latent infection. The definition of herpesvirus latency involves: (i) the presence of the viral genome in the nucleus of the infected cell (either as an episome or integrated in cellular chromosomes), (ii) reduced viral gene expression together with the absence of virion production, and (iii) the ability of latently infected cells to reactivate lytic viral replication either *in vivo* and/or *in vitro* (Lieberman, 2016). In addition, latent lifelong infection requires evasion mechanisms from the host immune response. Most alphaherpesviruses such as herpes simplex virus (HSV-1 or *human alphaherpesvirus 1 –* HHV-1) establish latency in non-dividing sensory neurons through maintenance of a quiescent episomal genome and expression of viral transcripts, in the absence of viral protein detection (Roizman and Whitley, 2013). In betaherpesviruses, myeloid cells such as macrophages are the main target of latent infection but the latency mechanisms in this subfamily have yet to be fully deciphered (Goodrum, 2016; Collins-McMillen et al., 2018). Gammaherpesviruses essentially establish latency in either B or T lymphocytes, although some species such as *bovine gammaherpesvirus 4* (BoHV-4) seem to infect cells of the monocyte/macrophage lineage (Machiels et al., 2011, 2013). The mechanisms regulating latency establishment, maintenance of such a dormant infection in actively dividing cells, and how gammaherpesviruses escape the immune system of the infected host have been thoroughly studied (Stevenson and Efstathiou, 2005; Blake, 2010; Barton et al., 2011; White et al., 2012; Lieberman, 2013; Schuren et al., 2016; Dong et al., 2017; Ueda, 2018).

Most significant advances in the understanding of herpesvirus latency mechanisms have been identified in gammaherpesviruses, which can probably be explained by the fact that one major latency protein, named the “*genome maintenance protein*” (GMP): (i) is encoded by the genome of all described gammaherpesvirus species, (ii) is expressed during latent infection, (iii) regulates the maintenance of viral episomes in actively dividing lymphocytes through tethering the viral genome to cellular chromosomes, and (iv) evades immune detection (Verma et al., 2007; Frappier, 2015). The main objective of this review is to briefly summarize the importance of gammaherpesvirus infections and how GMPs maintain viral episomes in infected lymphocytes, before focusing on a more detailed description of the mechanisms mediated by GMPs to escape immune surveillance, in particular CD8^+^ cytotoxic T cells (CTLs), during latent infection.

## The Subfamily *Gammaherpesvirinae*

Based on genomic and biological characteristics, gammaherpesviruses have been classified into four genera: the *Lymphocryptovirus* genus, the *Rhadinovirus* genus, the *Percavirus* genus and the *Macavirus* genus (Davison et al., 2009). Lymphocryptoviruses mainly infect human and non-human primates, and include one of the two gammaherpesviruses infecting humans: Epstein-Barr virus (EBV or *human gammaherpesvirus 4* – HHV-4) (Jha et al., 2016). Rhadinoviruses also infect human and non-human primates, and include the second human gammaherpesvirus, Kaposi’s sarcoma-associated herpesvirus (KSHV or *human gammaherpesvirus 8* – HHV-8) (Li et al., 2017). In addition to KSHV and viruses infecting Old World primates such as macaques, gorillas and chimpanzees, some rhadinoviruses also infect New World monkeys. Two examples are *saimiriine gammaherpesvirus 2* (or SaHV-2) also known as herpesvirus saimiri (HVS); and *ateline gammaherpesvirus 3*, which infects spider monkeys (Damania and Desrosiers, 2001). In addition to viruses infecting primates, rhadinoviruses also include a number of viral species infecting other mammalians (Davison et al., 2009). For instance, *murid gammaherpesvirus 4* (MuHV-4), also referred to as murine gammaherpesvirus 68 (MHV-68) is a natural pathogen of the yellow-necked field mouse (Ehlers et al., 2007) and is largely used in the laboratory mouse (*Mus musculus*). Furthermore, *Bovine herpesvirus 4* (BoHV-4) infection is prevalent in cows while this virus is thought to have originally evolved in another *Bovinae*, the African buffalo (*Syncerus caffer*) (Dewals et al., 2005). Macaviruses are viruses infecting ruminants and are mainly associated with a lymphoproliferative disease named malignant catarrhal fever (MCF). Among these, *alcelaphine gammaherpesvirus 1* (AlHV-1) and *ovine gammaherpesvirus 2 (*OvHV-2) naturally infect wildebeest (*Connochaetes taurinus*) and sheep (*Ovis aries*). They are responsible for wildebeest-derived and sheep-associated MCF in ruminants, respectively. The genus *Percavirus* is less well-defined and includes virus species infecting horses or mustelids.

The importance of latent infection by gammaherpesviruses is evident, both in term of lifelong persistence and related clinical diseases. The majority of epidemiological and clinical data comes from human gammaherpesviruses, although some veterinary viruses also induce latency-associated malignancies. EBV infects >90% of the human population, with seroconversion occurring early during childhood (Henle et al., 1969; Andersson, 2000). Whereas EBV latent infection is mostly asymptomatic, a number of clinical manifestations are associated with EBV infection. Beside infectious mononucleosis when primary infection occurs during adolescence (Callan et al., 1996), EBV is further associated with malignancies including Burkitt’s and Hodgkin’s lymphomas and other types of cancers (Rezk et al., 2018). In addition, EBV infection has been positively correlated with multiple sclerosis (MS). Nonetheless, the exact mechanisms and the causative role of EBV in MS induction remain open for investigation (Levin et al., 2010; Munger et al., 2011; Pender, 2011; Pakpoor et al., 2013; Moreno et al., 2018). The prevalence of KSHV is more variable and ranges from 5 to 50% depending on regions across the world. Like EBV, KSHV infection is in general asymptomatic but can be responsible for severe malignancies such as Kaposi’s sarcoma in immunocompromised patients but also other cancers such as multicentric Castlemans’s disease or primary effusion lymphoma (Li et al., 2017). Just like EBV and KSHV, animal gammaherpesviruses are also extensively studied, either for their veterinary importance or used as experimental models to study the biology of gammaherpesvirus infection *in vivo*. The former category includes MCF-inducing AlHV-1 that is responsible for the induction of a deadly peripheral T cell lymphoma-like disease in cattle caused by latently infected CD8^+^ T lymphocytes (Dewals et al., 2008, 2011; Dewals and Vanderplasschen, 2011; Palmeira et al., 2013). MuHV-4 and SaHV-2 are two examples of animal gammaherpesviruses of the latter category. Whereas the functions of gammaherpesvirus GMPs have been extensively investigated for EBV and KSHV in cell culture *in vitro*, animal gammaherpesviruses have provided important insights on the role of GMPs during *in vivo* infection.

## Genome Maintenance Proteins and Their Roles During Gammaherpesvirus Infection

After primary infection of target cells, gammaherpesviruses enter the lytic cycle that leads to production of viral particles and cell death. However, and depending on the infected cell type, the latent phase of the infection is in general rapidly established and is accompanied with the production of latency transcripts, including GMP. All sequenced gammaherpesviruses encode a predicted GMP (Table 1). In lymphocryptoviruses, GMPs are encoded by open reading frame (ORF) BKRF1 and are named according to Epstein-Barr virus (EBV) nuclear antigen 1 (EBNA-1). The GMPs in *Rhadinovirus, Percavirus*, and *Macavirus* genera are encoded by ORF73 and can be named after KSHV latency-associated nuclear antigen 1 (LANA-1). GMPs are DNA-binding proteins able to bind sequences within the viral genome while at the same time interacting with cell chromosome-associated proteins, to ensure partitioning to daughter cells during mitosis. Early studies have already demonstrated that GMPs, such as EBNA-1 and LANA-1, are essential for episome persistence (Ballestas et al., 1999; Ballestas and Kaye, 2001; Sears et al., 2003). Similar data have been generated for SaHV-2 and MuHV-4 for which it has been demonstrated that the gene ORF73 is required for efficient establishment of latency (Fowler et al., 2003; Calderwood et al., 2005). Likewise, deletion from the BoHV-4 genome of ORF73 impaired viral persistence in a macrophage cell line *in vitro* and *in vivo* in the rabbit model (Thirion et al., 2010). In addition, the ORF73-encoded protein of strain H26-95 of *macacine gammaherpesvirus 5* was shown to bind to the viral episome and to be essential for establishment of latency (DeWire and Damania, 2005; Wen et al., 2009). The deletion of ORF73 from the genome of AlHV-1 also rendered AlHV-1 unable to persist and induce MCF *in vivo*, whereas impairment of its expression did not affect viral lytic replication (Palmeira et al., 2013). To enable partitioning in proliferating cells and avoid losing the episomal genomes in the cytoplasm, GMPs bind viral episomes to host chromosomes. Tethering of viral episomes to host DNA is accomplished by the ability of GMPs to simultaneously bind to several chromosome-associated proteins, other cellular components of the mitotic apparatus and specific viral DNA sequences (Yates et al., 1984; Cotter and Robertson, 1999; Shire et al., 1999; Cruickshank et al., 2000; Wu et al., 2000; Ballestas and Kaye, 2001; Cotter et al., 2001; Kapoor and Frappier, 2003; Verma and Robertson, 2003; Barbera et al., 2004, 2006; Calderwood et al., 2004; Waldmann et al., 2004; Kapoor et al., 2005; You et al., 2006; Kelley-Clarke et al., 2007; Habison et al., 2012; Verma et al., 2013; Gupta et al., 2016).

**Table 1:** Gammaherpesvirus genome maintenance proteins based on functional evidence and/or sequence prediction.

Genus/species	Common name (common abbreviation)	GMP	Size (amino acids)	Central repeat (size)^∗^	Central repeat content^†^	Accession number
**Lymphocryptovirus**						
*Callitrichine gammaherpesvirus 3*	Marmoset herpesvirus	ORF39	327	– (NA)	–	NP_733892
*Human gammaherpesvirus 4*	Epstein-Barr virus (EBV)	EBNA1	641	+ (239)	GA	YP_401677
*Macacine gammaherpesvirus 4*	Rhesus lymphocryptovirus (rhLCV)	rhEBNA1	511	+ (47)	GSA	YP_067973
*Papiine gammaherpesvirus 1*	Herpesvirus papio	baEBNA1	476	+ (49)	GSA	AAA66373
**Rhadinovirus**						
*Ateline gammaherpesvirus 3*	Herpesvirus ateles strain 73 (AtHV-3)	ORF73	447	+ (157)	DG(E)	NP_048045
*Bovine gammaherpesvirus 4*	Bovine herpesvirus 4, Movar virus, V. test virus	ORF73 (boLANA)	243	– (NA)	–	NP_076567, AEL29819
*Cricetid gammaherpesvirus 2*	Rodent herpesvirus Peru	RHP73	294	– (NA)	–	YP_004207909
*Human gammaherpesvirus 8*	Kaposi’s sarcoma-associated herpesvirus (KSHV)	LANA1	1129	+ (585)	Acidic	YP_001129431
*Macacine gammaherpesvirus 5^‡^*	Rhesus rhadinovirus	ORF73 (rhLANA)	1071^§^	+/- (521)	Acidic	ABH07414
*Murid gammaherpesvirus 4*	Murine gammaherpesvirus 68 (MHV68)	ORF73 (mLANA)	314	– (NA)	–	NP_044913
*Murid gammaherpesvirus 7*^¶^	Wood mouse herpesvirus	ORF73	327	– (NA)	–	ACY41142
*Saimiriine gammaherpesvirus 2*	Herpesvirus saimiri (HVS)	ORF73 (sLANA)	501^#^	+ (265)	GE/EA	CAC84371
**Percavirus**						
*Equid gammaherpesvirus 2*	Equine herpesvirus 2 (EHV-2)	ORF73	985	+ (578)	Acidic	YP_009118179
*Equid gammaherpesvirus 5*	Equine herpesvirus 5 (EHV-5)	ORF73	996	+ (476)	Acidic	YP_009118462
**Macavirus**						
*Alcelaphine gammaherpesvirus 1*	Wildebeest-derived malignant catarrhal fever virus	ORF73 (aLANA)	1324	+ (986)	G(P)E	APB09566, ATI21957
*Alcelaphine gammaherpesvirus 2*	Topi herpesvirus	ORF73	1277	+ (913)	G(P)E	YP_009044454
*Ovine gammaherpesvirus 2*	Sheep-associated malignant catarrhal fever virus	ORF73 (oLANA)	495	+ (330)	G(P)E	YP_438196

*^∗^Presence of a central repeat (CR) domain is indicated. The size of the present CR domain is given for the prototypic sequence for which the genbank accession number is provided.*

*^†^Information on the composition of most represented amino acid residues or the presence of an acidic domain without clear consensus repeat in the CR is given. D, aspartic acid; E, glutamate; G, glycine; P, proline; S, serine.*

*^‡^Macacine gammaherpesvirus 5 are a group of closely related viruses of Old and New World non-human primates that are classified in two lineages within the Rhadinovirus genus, RV1 and RV2 (Damania and Desrosiers, 2001).*

*^§^Only RV1 viruses possess a CR domain. The given accession number is from strain M78114.*

*^¶^Murid gammaherpesvirus 7 is phylogenetically related to MuHV-4 and Brest herpesvirus. No GMP sequence is available for the latter (Chastel et al., 1994; Hughes et al., 2010).*

*^#^The GMP of SaHV-2 is given for strain C488*.

**FIGURE 1:**
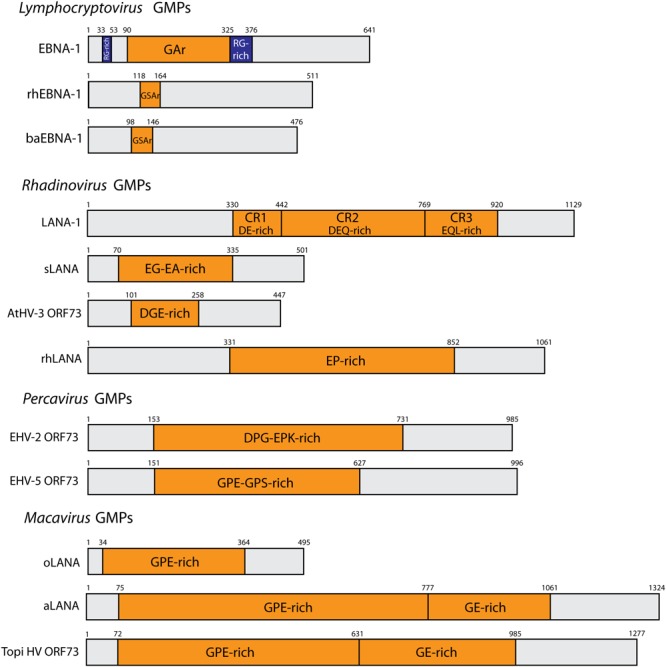
Schematic representation of representative gammaherpesvirus GMPs. N- and C- terminal domains are separated by a central amino acid repeat domain (CR), highlighted in orange. Repeat residues are indicated. The RG-rich regions of EBNA-1 are depicted in blue. Genus *Lymphocryptovirus*: EBNA-1 (EBV, strain B95.8), rhEBNA1 (rhLCV, strain LCL8664) and baEBNA-1 (baLCV, strain S594). Genus *Rhadinovirus*: AtHV3 ORF73 (strain 73), LANA-1 (KSHV, strain JK-18), sLANA (SaHV-2, strain C488), rhLANA (M78114). Genus *Percavirus*: EHV-2 ORF73 (strain 86/87), EHV-5 (strain 2-141/67). Genus *Macavirus*: oLANA (OvHV-2, strain BJ1035), and aLANA (AlHV-1, strain C500).

Genome maintenance proteins are nuclear proteins with very little sequence similarity among gammaherpesviruses, even though GMPs within one genus show higher sequence similarity. The C-terminal region shows the highest degree of sequence similarity and is involved in DNA-binding (Tellam et al., 2012). Regarding protein primary structure, most GMPs contain a central repeat (CR) domain composed of repeated amino acid motives that are divergent between gammaherpesvirus species. Interestingly, the size of the different GMPs greatly varies due to the presence of the CR domain (Figure 1). However, such variation in size does not seem to alter its role in maintaining the viral genome and the CR domain appears to be dispensable for genome maintenance properties with some gammaherpesviruses being devoid of a CR domain, such as MuHV-4 or BoHV-4 (Lomonte et al., 1995; Bennett et al., 2005). In addition to genome-maintenance functions, studies performed essentially on EBV EBNA-1 and KSHV LANA-1 have revealed additional roles for GMPs during latency and latency-associated diseases. GMPs are involved in initiating viral DNA replication during latency to generate sufficient copies of viral episomes prior to cell division (Wysokenski and Yates, 1989; Harrison et al., 1994; Yates et al., 2000; Ballestas and Kaye, 2001; Cotter et al., 2001; Stedman et al., 2004; Wong and Wilson, 2005; Verma et al., 2006; Lu et al., 2012), modulating viral gene expression to promote latency and repress reactivation (Gahn and Sugden, 1995; Evans et al., 1996; Schafer et al., 2003; Lu et al., 2006; Sivachandran et al., 2012), promoting tumorigenesis (Radkov et al., 2000; Humme et al., 2003; Altmann et al., 2006; Cai et al., 2006, 2012), and evading the immune system.

## Genome Maintenance Proteins and Their Immune Evasion Properties

**FIGURE 2:**
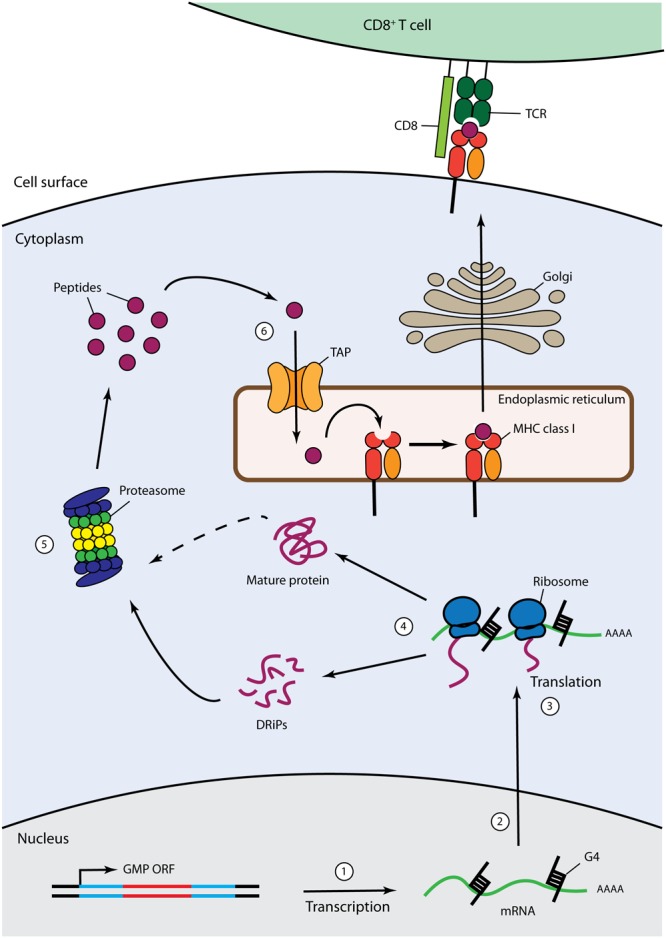
*Cis*-acting immune evasion of MHC Class I antigen presentation of gammaherpesvirus GMPs. The MHC class I antigen presentation pathway is depicted with described GMP-mediated *cis*-acting immune evasion mechanisms. Cytoplasmic endogenously expressed viral proteins are degraded by the proteasome into antigenic peptides that are then translocated from the cytosol to the endoplasmic reticulum (ER) through the transporter for antigen processing (TAP). Then, antigenic peptides are loaded on MHC class I molecules to form MHC-I-peptides complexes that are subsequently exported to the cell surface through the Golgi apparatus for recognition by CD8^+^ cytotoxic T lymphocytes. GMPs have been demonstrated to inhibit this process through various mechanisms: (1) sLANA and aLANA were shown to decrease their own steady-state RNA levels; (2) EBNA-1 can inhibit pre-mRNA processing of the primary EBNA-1 transcript; (3) structural constraints, such as G-quadruplexes (G4), contained in aLANA and EBNA-1 mRNA rather than protein sequences can regulate self-translation; (4) EBNA-1, aLANA, LANA-1, sLANA, and mLANA are able to induce retardation of self-translation; (5) EBNA-1, mLANA, and LANA-1 were shown to be protected from proteasomal degradation; (6) LANA-1 was reported to hold an inhibitory effect prior to translocation of its own cytoplasmic peptides into the ER.

To ensure lifelong latency, gammaherpesviruses must maintain their genomes in dividing cells and remain undetected by virus-specific CD8^+^ CTLs. Thus, the GMPs must overcome the dilemma of efficiently maintaining viral episomes within infected cells while, at the same time, evading immune surveillance. Viral proteins are expressed endogenously within cells and are thus degraded by the proteasome into antigenic peptides before being translocated from the cytosol to the endoplasmic reticulum (ER) and loaded on major histocompatibility class I (MHC class I) molecules to form MHC-I-peptide (MHCp) complexes. MHCp complexes are exported to the cell surface for recognition by CD8^+^ cytotoxic T lymphocytes (CTLs) (Blum et al., 2013) (Figure 2).

The main source of viral antigens was previously thought to uniquely arise from the turnover of mature proteins. However, more recent studies highlighted an alternate hypothesis regarding the origin of MHC class I-restricted viral peptides, pointing to a major role of defective ribosomal products (DRiPs) as the main source of antigenic peptides during viral infection (Yewdell et al., 1996; Yewdell, 2011). DRiPs are translational products derived from prematurely terminated or misfolded polypeptides. Prioritizing DRiPs as the main source of antigenic peptides is believed to provide opportunities for the immune system to rapidly detect an active viral infection and thus optimize immune surveillance (Anton and Yewdell, 2014; Wei and Yewdell, 2018). Nevertheless, data supporting this hypothesis are still limited and further studies are necessary to exactly quantify whether MHC class I antigen presentation can be attributed to DRiPs of newly synthesized proteins. Indeed, the MHC class I-presented peptides seem to come from both short-lived and stable mature proteins depending on the origin of proteins and the biological status of the cell environment (Rock et al., 2014, 2016). Although autophagy is in some key aspects involved in the induction of adaptive immunity and control of some viral infections (Paludan et al., 2005), it appears that gammaherpesviruses have rather evolved to develop strategies to co-opt autophagy for viral benefit, during the lytic cycle but also during latency. However, GMPs do not seem to be directly involved in such mechanisms [see recent review (Lussignol and Esclatine, 2017)].

Gammaherpesviruses have evolved to acquire different strategies to escape the immune response. These mechanisms have been extensively reviewed in the past (Stevenson and Efstathiou, 2005; Means et al., 2007; Blake, 2010; Feng et al., 2013; Hu and Usherwood, 2014; Ressing et al., 2015; Sorel and Dewals, 2016; Zuo et al., 2017). The significant downregulation of viral gene expression during latent infection contributes to immune evasion, with viral gene expression being restricted to non-coding RNAs and latency proteins, including GMPs. GMPs must indeed be expressed in infected cells due to their key functions during long-term latency but at the same time need to remain hidden from the immune system. In order to evade CTL recognition of latently infected cells, all studied gammaherpesvirus GMPs have evolved to put into place immune evasion mechanisms consisting of the inhibition of their own presentation in the context of MHC class I on the cell surface, a mechanism that has been termed “*cis*-acting immune evasion” (Figure 2). Pioneer work came from studying EBV GMP where the CR domain could directly be involved in self-inhibition of antigen presentation (Levitskaya et al., 1995). Although most GMPs encoded by gammaherpesviruses contain a CR domain and are generally involved in the described *cis*-acting immune evasion mechanism, GMPs encoded by MuHV-4 (mLANA) or BoHV-4 (boLANA) do not have a CR domain (Lomonte et al., 1995; Bennett et al., 2005). While no data are available for immune evasion mechanism by boLANA, mLANA was able to inhibit self-presentation in MHC class I despite its lack of a CR domain (Bennett et al., 2005). Intriguingly, despite the conserved functions of gammaherpesviruses GMPs, the peptidic sequence in their repeat regions differs to a great extent from one ortholog to another. In contrast, comparative mRNA sequence analysis revealed that the internal repeat regions of GMP mRNAs displayed high nucleotide sequence similarities (Tellam et al., 2012). The results detailed in the next sections suggest that GMPs have evolved to adopt various strategies depending on the viral species, in order to achieve the ultimate goal consisting of the inhibition of their own presentation by MHC class I.

## Genus *Lymphocryptovirus*

### Epstein-Barr Virus (EBV)

Epstein-Barr virus EBNA-1 is one of the most studied GMPs with regards to its different functional aspects, from genome maintenance (Yates et al., 1984, 1985), to *cis*-acting immune evasion. Early studies have demonstrated that EBNA-1 is able to prevent MHC class I-restricted self-peptide presentation in *cis* to CTLs through a mechanism involving its CR domain (GAr) (Levitskaya et al., 1995). The GAr domain is a region rich in glycine (G) and alanine (A) residues, which corresponds to 239 aa in strain B95.8 of EBV (Baer et al., 1984). Although its size can vary based on the strain, all isolates contain a GAr region (Falk et al., 1995). In order to decipher the immune evasion mechanism driving this effect, several studies have shown that the GAr domain provides increased stability to the EBNA-1 protein by inhibiting proteasome-mediated degradation (Levitskaya et al., 1997; Sharipo et al., 1998; Heessen et al., 2002, 2003; Hoyt et al., 2006; Coppotelli et al., 2011). In these studies, increased CTL responses could be induced with EBNA-1ΔGAr recombinant proteins, where the GAr was removed. Using conventional chromium-51 cytotoxicity assays, MHC-tetramer stains and/or peptide restimulation, it appeared that removal of the GAr domain resulted in increased presentation of a model T-cell epitope inserted into the GMP backbone but also increased presentation of T-cell epitopes of EBNA-1 itself, including the HLA-B^∗^35:01-restricted CTL epitope (HPVGEADYFEY). Despite the potential self-protection of EBNA-1 from MHC class I antigen presentation, several studies found that EBNA-1-specific CTLs exist in EBV-seropositive patients (Blake et al., 1997, 2000; Subklewe et al., 1999; Sim et al., 2013). Whereas inhibition of proteasome degradation was initially suggested, other reports found that GAr would more likely self-inhibit protein translation efficiency to reduce presentation of CTL epitopes from EBNA-1. These latter observations led to the conclusion that the GAr domain could inhibit the production of translation-dependent DRiPs. However, it remains unresolved whether control of the production of DRiPs during *de novo* translation is the only mechanism explaining EBNA-1 *cis*-acting immune evasion. Indeed, additional data contradicted the direct implication of the GAr domain in protecting EBNA-1 from proteasome degradation and increased protein stability (Yin et al., 2003; Lee et al., 2004; Tellam et al., 2004, 2007a; Voo et al., 2004; Daskalogianni et al., 2008).

What is clear from the studies investigating *cis*-acting immune evasion of EBNA-1 is that the GAr-mediated self-inhibition of antigen presentation to CTLs is not absolute. Indeed, although reduced in presence of GAr, EBNA-1 can be immunogenic and lead to the development of EBNA-1-specific CTLs in humans (Blake et al., 1997, 2000; Subklewe et al., 1999; Sim et al., 2013), but also in a mouse model where EBNA-1 was transduced using an adenovirus expression vector (Tellam et al., 2014). Recent advances have put forward a hypothesis to explain how the GAr domain is able to reduce self-translation efficiency. Studies which investigated mRNA translation of EBNA-1 suggested that the nascent GAr peptide alone was able to delay the assembly of the translation initiation complex mRNA, therefore reducing mRNA translation (Apcher et al., 2009, 2010). However, more recent reports suggested that EBNA-1 mRNA structure itself rather than the GAr peptidic sequence could regulate EBNA-1 protein translation (Tellam et al., 2008). Further findings supported this hypothesis by demonstrating that mRNA sequence could regulate the level of self-synthesis and antigen presentation of EBNA-1 *in vitro* through the GAr domain (Tellam et al., 2012). These authors highlighted the fact that the GAr domain, but also most GMP CR domains, display a nucleotidic sequence bias with enrichment of purines that is associated with reduced efficiency of protein translation. The role of purine-rich domains was demonstrated when replacement of the third base position of codons by pyrimidines led to increased translation of the protein and CTL activation (Tellam et al., 2008, 2014). Moreover, generating frameshifts in the EBNA-1 GAr internal repeat sequence to create alternate peptidic repeats had no effect on the *cis*-acting immune evasion (Tellam et al., 2012). Indeed, EBNA-1 frameshift mutants expressing GQE-rich or GRS-rich repeats could inhibit the presentation of a linked model epitope with an efficacy similar to native EBNA-1. Shortly after, the same group highlighted a key role played by clusters of unusual structural elements within the EBNA-1 mRNA sequence, named G-quadruplexes (G4), in the modulation of protein synthesis (Murat et al., 2014). G4 are secondary structures of nucleic acids that form within G-rich DNA or RNA sequences (Murat and Tellam, 2015). Four guanine bases can associate through hydrogen bonding to form a guanine tetrad and two or more guanine tetrads can stack on top of each other to constitute a G4 structure (Metifiot et al., 2014). Globally, these structures are present in telomeres, promoters, and gene bodies where they perform important regulatory roles in diverse biological processes including replication, transcription and translation (Rhodes and Lipps, 2015). Bioinformatics analysis of the EBNA-1 mRNA sequence revealed the presence of multiple putative G4 structures within the GAr domain (Murat et al., 2014). These authors further demonstrated that destabilization of G4 structures using antisense oligonucleotides led to an increase of EBNA-1 mRNA translation (Murat et al., 2014). To go further, as mentioned above, a modification of codon usage to reduce the purine bias in GAr resulted in reverted *in vivo* MHC class I epitope presentation and early priming of CD8^+^ T cells (Tellam et al., 2014). The mechanism underlying this effect was suggested to be determined by a capacity of G4 structures present in GAr to alter the association of ribosomes with EBNA-1 mRNA by inducing premature termination and/or ribosome stalling, therefore impeding protein translation (Murat et al., 2014). Nonetheless, whether G4 structures are present in all gammaherpesvirus GMPs and involved in self-inhibition of protein translation for immune evasion, needs to be further elucidated. Interestingly, the generation of memory T cell response was not affected by the codon-modification within the GAr domain (Tellam et al., 2014). These results were of high interest as they reported that promoting CTL priming against EBNA-1 through impairment of the GAr-dependent *cis*-acting immune evasion mechanisms could result in a more rapid CTL response and the establishment of efficient immune memory. In addition to translation regulation, previous studies have also established that EBNA-1 could act at the transcriptional level through inhibition of pre-mRNA processing of the primary EBNA-1 transcript (Yoshioka et al., 2008).

### Other Lymphocryptoviruses

Studies on the GMPs encoded by baboon lymphocryptovirus (baLCV) and rhesus lymphocryptovirus (rhLCV), namely baEBNA-1 and rhEBNA-1, respectively, provided conflicting data. Indeed, the first results suggested that both the ba- and rhGSAr domains were not able to prevent MHC class I restricted peptide presentation in *cis* (Blake et al., 1999), whereas a second study showed that rhEBNA-1-specific CTLs expanded *in vitro* from rhLCV-infected animals failed to recognize endogenously expressed rhEBNA-1 (Fogg et al., 2005). A more recent study provided data supporting the hypothesis whereby rhEBNA-1 and baEBNA-1 proteins do not possess *cis-*acting immune evasion properties (Tellam et al., 2007b). Both proteins were translated at a higher rate than EBV EBNA-1 with no effect of deletion of the GSAr domains on translation rates and the rhGSAr domain could not avoid *cis*-acting presentation of a model epitope. A potential explanation for the adversarial result regarding the lack of recognition of rhEBNA-1 by the rhEBNA-1-specific CTLs reported in a study by Fogg and collaborators, could be that the specific clones isolated were of low affinity against the GMP (Blake, 2010).

## Genus *Rhadinovirus*

### Kaposi’s Sarcoma-Associated Herpesvirus (KSHV)

LANA-1 is also able to act in *cis* to inhibit MHC class I-restricted epitope presentation to CTLs through involvement of the CR domain (Zaldumbide et al., 2007). LANA-1 is subdivided into three domains based on the peptidic sequence, with imperfect repeats: CR1 (aa 330–442) is a DE-rich region, CR2 (aa 442–768) is a DEQ-rich region, and CR3 (aa 769–920) consists of an EQL-rich region (Figure 1). Interestingly, the size of the CR domain varies between different KSHV strains or isolates (Gao et al., 1999). While a junctional domain between LANA-1 CR2 and CR3 has been mapped to contribute to retardation of translation and inhibition of proteasomal degradation of LANA-1 (Kwun et al., 2007), neither the CR2 nor CR3 domains were found to be involved in the inhibition of peptide presentation (Kwun et al., 2011). These data suggested that, in contrast to EBNA-1, the mechanism combining protection of LANA-1 from proteasomal degradation and reduction in the DRiPs generation level is not sufficient to block peptide presentation on MHC class I. Another notifiable difference with EBNA-1 is that the retardation of LANA-1 translation seems to be due to CR amino acid sequence rather than to the nucleotide level. Indeed, the introduction of a stop codon between CR2 and CR3 resulted in increased translation (Kwun et al., 2007). This observation is of importance considering the high degree (about 50% for CR1 and CR2, and about 70% for CR3) of similarity between EBNA-1 and LANA-1 in terms of nucleotidic sequence (Tellam et al., 2012). Conversely, CR1 has been implicated in LANA-1 *cis*-acting immune evasion through an apparent inhibitory effect prior to translocation of cytoplasmic peptides into the ER (Kwun et al., 2011). Here, fusion of a signal peptide to LANA-1 led to efficient processing of the protein for MHC Class I presentation. However, the presence or absence of CR1 had no effect on protein translation or proteasomal degradation. Using interferon-dependent induction of proteasomal degradation and proteasome inhibitor MG132, Kwun and collaborators further highlighted that LANA-1 is processed for MHC I presentation through the canonical proteasome pathway with little contribution of autophagy (Kwun et al., 2007, 2011). Thus, LANA-1 seems to have evolved to adopt immune evasion mechanisms that differ from EBNA-1, despite having conserved nucleotide sequence similarities. Although G4 structures have been involved in KSHV DNA replication and episomal persistence (Madireddy et al., 2016), it remains unappreciated whether G4 structures are present in LANA-1 or not, as demonstrated for EBNA-1. EBNA-1-specific CD8^+^ CTLs could be identified in patients (Blake et al., 1997, 2000; Subklewe et al., 1999; Sim et al., 2013). Likewise, several studies have identified LANA-1-specific CD8^+^ T-cell responses in KSHV seropositive subjects, highlighting the premise that the self-protection of GMPs against MHC class I-restricted epitope presentation is not absolute (Brander et al., 2002; Woodberry et al., 2005; Bihl et al., 2007; Lepone et al., 2010). Nonetheless, to our knowledge, no LANA-1-specific T cell clone is available to be tested *in vitro*.

### Other Rhadinoviruses

Infection of squirrel monkeys with SaHV-2 results in asymptomatic latency in T lymphocytes. However, co-species transmission to New World non-human primates can lead to the development of acute T-cell lymphomas (Fickenscher and Fleckenstein, 2001). Interestingly, SaHV-2 can induce transformation of human and rabbit T lymphocytes (Fleckenstein and Ensser, 2004). The transforming capability of SaHV-2 has been identified to be mainly driven by two viral proteins termed Stp and Tip (Fleckenstein and Ensser, 2007). SaHV-2 infection of T lymphocytes is associated with episomal maintenance in absence of production of viral particles and the GMP encoded by SaHV-2 (sLANA) has been demonstrated to be essential for episomal maintenance (Calderwood et al., 2005). The CR domain of sLANA varies between strains, with a EG-rich domain of 15 aa in strain A11 or of 111 aa in strain C488; and a EA-rich region of 147 and 132 aa in both strains, respectively. One study thoroughly investigated the role of sLANA in evading CTL recognition (Gao et al., 2009). In this study, Gao and collaborators demonstrated that sLANA could reduce the MHC class I presentation of the linked-OT1 epitope SIINFEKL (Gao et al., 2009). The authors further observed that the steady-state levels of sLANA protein were reduced due to the presence of the CR domain rich in EG and EA residues, an observation that could be explained by a decrease in the steady-state levels of sLANA mRNA. Unexpectedly, the CR domain was not responsible for an increased turnover of sLANA mRNA but for a better stability over time of sLANA mRNAs compared to constructs deleted of the EG-EA repeat. Moreover, the authors showed that neither protein stability nor the efficiency of protein translation were influenced by the CR region, which revealed significant differences compared to both EBNA-1 and LANA-1. Finally, a single copy of the motif EEAEEAEEE, which is present multiple times in the EA-rich domain of two strains of SaHV-2, was shown to be sufficient to inhibit MHC class I-restricted antigen presentation when fused in frame with the sequence of the heterologous ovalbumin protein (Gao et al., 2009). The mechanism underlying this effect was suggested to be due to both stabilization of mRNA and repression of self-transcription. Thus, the presence of the EA-rich region could potentially influence the total amount of translated sLANA and protein synthesis efficiency, which in turn could potentially reduce the generation of DRiPs although this aspect has not been directly addressed.

MuHV-4 infects and persists in the laboratory mouse. Following primary infection, usually experimental intranasal or intra-tracheal infection, MuHV-4 replicates in epithelial cells and macrophages before reaching secondary lymphoid organs where the virus is maintained as episomal genomes in memory B lymphocytes (Barton et al., 2011; Gillet et al., 2015). MuHV-4 GMP (mLANA) has been demonstrated to be essential for episome maintenance *in vitro* but also *in vivo* as shown using recombinant strains of MuHV-4 impaired for the expression of mLANA (Fowler et al., 2003; Forrest et al., 2007; Habison et al., 2012). Among gammaherpesvirus GMPs, mLANA is probably one of the most intriguing proteins. Indeed, despite a lack of CR domain, mLANA has retained the ability of its orthologs to act in *cis* to self-inhibit MHC class I antigen presentation through a region mapped to amino acids 170–220 of mLANA (Bennett et al., 2005). This region was shown to be able to decrease the steady-state levels of mLANA protein while at the same time contributing to enhancing protein stability and protection from proteasomal degradation (Bennett et al., 2005), similar to EBNA-1. However, the exact mechanisms of action have not been fully deciphered and it remains unclear how protein translation efficiency and potential G4 structures could be involved. MuHV-4 provides an invaluable model to study gammaherpesvirus infection *in vivo*, including viral pathogenesis and latency (Barton et al., 2011). Using recombinant strains of MuHV-4 expressing model T-cell epitopes in tandem under the control of ORF73, Bennett and collaborators used an internal ribosome entry site following the ORF73 coding sequence to bypass the *cis*-acting evasion of ORF73 and to force expression of a tandem sequence of three T-cell epitopes *in vivo*. By doing so, latently-infected cells could express, in *trans*, CTL epitopes encoded by the ORF73 mRNA. Using this recombinant virus, infected mice showed a critical MHC class I-restricted and CTL-dependent reduction in viral latency, demonstrating that *trans-*acting immune evasion could not inhibit peptide presentation to CTLs during latency but rather indirectly suggests that *cis*-acting evasion by the GMP is critical for normal establishment of long-term latency *in vivo*. This study was the first to tackle the immune evasion mechanisms of GMPs *in vivo*. Interestingly, three recent studies used chimeric MuHV-4 recombinant viruses where mLANA was replaced by functional KSHV LANA-1 (Gupta et al., 2017; Habison et al., 2017; Pires de Miranda et al., 2018). These studies demonstrated the ability of LANA-1-expressing chimeric MuHV-4 to be maintained and establish latency *in vivo*, although at lower levels compared to wildtype MuHV-4. These studies are encouraging for future prospects to further investigate the role of immune evasion mechanisms *in vivo* that are directly mediated by LANA-1 or even other related GMPs.

## Genus *Percavirus*

Numerous clinical syndromes have been identified in equid species in association with EHV-2 or EHV-5 infection. However, true evidence for causal implication in the described diseases remain elusive (Marenzoni et al., 2015), with the exception of pulmonary fibrosis induced by EHV-5 infection (Williams et al., 2013). The full sequence of only two members of the *Percavirus* genus has been recently obtained. Although originally thought to not express a GMP (Telford et al., 1995), a recent report identified ORF73-encoding GMPs in two strains of *equid gammaherpesvirus 2* (EHV-2), including the initially sequenced strain, and one strain of *equid gammaherpesvirus 5* (EHV-5) (Wilkie et al., 2015). Whereas strain 86/67 of EHV-2 expresses a GMP of 985 amino acids, strain G9/92 expresses a 949-aa GMP. Strain 2-141/67 of EHV-5 encodes a GMP of 996 residues. Although GMPs of EHV-2 and EHV-5 contain a predicted CR domain, no data is currently available on a potential *ci*s-acting immune evasion. More studies need be performed to uncover the role of EHV-2 and EHV-5 GMPs in infection of equids with these viruses.

## Genus *Macavirus*

All sequenced macaviruses encode a GMP but only the role of AlHV-1 GMP (aLANA) has recently been investigated experimentally (Palmeira et al., 2013; Sorel et al., 2017). AlHV-1 infects and persists in wildebeest asymptomatically and one can assume that the entire population of free-ranging wildebeest are infected. However, upon reactivation events, AlHV-1 can be transmitted to a range of phylogenetically related ruminant species, like cattle. In these susceptible species, AlHV-1 induces MCF that ultimately leads to the death of the infected animal. In both wildebeest and cattle, AlHV-1 establishes latency but results either in true quiescent/latent infection (in wildebeest) or latency-associated lymphoproliferation of CD8^+^ T lymphocytes (in cattle). During AlHV-1-associated MCF, aLANA is highly expressed (Palmeira et al., 2013). Thus, an adaptive immune response is likely induced against the protein, potentially including CD8^+^ CTLs that are specific to aLANA-derived antigenic peptides. However, if such a putative response exists, it fails to be protective as MCF-susceptible animals ultimately develop MCF and die upon infection. In our recent study, aLANA was shown to have acquired *cis*-acting immune evasion properties similarly to its orthologs (Sorel et al., 2017). In particular, this immune evasion mechanism was shown to be mediated through the CR domain of aLANA that is rich in G and E residues (termed GE). Importantly, the inhibitory properties of GE could be transferred to a heterologous protein such as enhanced green fluorescent protein, which is consistent with the data obtained with the EA-rich domain of sLANA (Gao et al., 2009). Mutant constructs expressing aLANA deleted for the GE-rich domain exhibited similar protein and mRNA turnover suggesting that GE inhibits proteasome-dependent antigen presentation through a mechanism that does not involve protein or mRNA degradation processes (Sorel et al., 2017). Although these data are consistent with the results obtained with sLANA (Gao et al., 2009), the internal region of several GMPs, including LANA-1 and EBNA-1, as well as the amino acid region 170–220 of mLANA were shown to mediate decreased protein turnover (Levitskaya et al., 1997; Bennett et al., 2005; Kwun et al., 2007). However, the CR2CR3 region of LANA-1 that was mapped to inhibit proteasomal degradation was, however, not found to be involved in the self-inhibition of antigen presentation (Kwun et al., 2011). Although the mechanisms involved in immune evasion are not necessarily shared by all gammaherpesvirus GMPs, these data are nonetheless strengthened by another study that revealed that the half-life of a polypeptide does not determine antigen presentation (Apcher et al., 2010). Thus, it can be suggested that protection of GMPs from proteasomal degradation might not be sufficient to block antigen presentation. The lack of aLANA GE resulted in increased protein expression levels due to a combination of enhanced translation efficiency and increased steady-state RNA levels (Sorel et al., 2017), which resulted in increased proteasome-dependent processing of aLANA for MHC-I presentation. This mechanism was, however, independent of autophagy, as treatments with the autophagy inducer rapamycin, or autophagy inhibitors chloroquine or 3-methyladenine, did not affect peptide presentation by MHC-I. Thus, these results suggested that the GE-rich domain could inhibit self-antigen presentation through regulation of both protein translation and RNA transcription levels, leading as a consequence to a decrease in DRiPs generation. Several related gammaherpesviruses were shown to have acquired mechanisms that lead to reduced steady-state protein levels of their respective GMPs (Tellam et al., 2007a; Yoshioka et al., 2008; Gao et al., 2009), resulting in a potential reduction of DRiPs production. Thus, targeting pathways leading to DRiPs production seems to be a valuable mechanism to ensure episome persistence during latency while avoiding detection by the immune system. Furthermore, replacing the native GE-rich region of aLANA by a synthetic codon-modified sequence, in order to reduce the purine bias in the mRNA sequence without modifying the protein sequence, similar to EBNA-1 GAr (Tellam et al., 2014), led to significantly enhanced antigen presentation and increased activation of antigen-specific CTLs in a mouse model of DNA immunization (Sorel et al., 2017). These results were suggestive of potential constraints, such as G4 structures, within native GE mRNA structure that could limit antigen presentation in a similar manner as EBNA-1 (Tellam et al., 2008; Murat et al., 2014). Then, mRNA constraints contained in the GE-rich domain of aLANA, rather than peptidic sequence, is likely responsible for CTL immune evasion. However, the GE-mediated *cis*-limitation of MHC class I antigen presentation of aLANA was further shown to be dispensable for the induction of MCF in the experimental rabbit model (Sorel et al., 2017). Indeed, a recombinant virus expressing a GE-deleted form of aLANA could induce MCF in rabbits in a similar manner to a wild type virus expressing aLANA. Although the viral-specific CTL response could not be monitored to determine the role of the GE-rich domain in the efficient priming of CTLs by aLANA *in vivo*, it clearly appears that aLANA-mediated *cis*-acting immune evasion is not determinant during MCF. While the mechanisms explaining this finding in the context of MCF remain to be identified, these results suggest that the immune evasion functions of aLANA are more likely to play a role in the context of lifelong infection of the natural host of AlHV-1, the wildebeest.

## Conclusion

All sequenced gammaherpesviruses encode a GMP that tethers viral genomes to the cellular chromosomes, ensuring even segregation of viral episomes in daughter cells during cell division (Blake, 2010). Besides their role in viral persistence, GMPs can also modify the cellular environment to promote cell immortalization and tumorigenesis in gammaherpesvirus-induced malignancies. Because of their essential roles in gammaherpesvirus latency, GMPs need to be expressed while remaining hidden from immune surveillance in the infected host. Evasion mechanisms of the cytotoxic T cell response through self-limitation of MHC class I antigen presentation constitute unique properties developed by GMPs to ensure gammaherpesvirus long-term persistence. Importantly, more questions need to be addressed for a complete understanding of how GMPs successfully achieve both viral persistence and escape of the CTL response. Such future understanding is of interest to develop potential treatments to target and efficiently disrupt latency. Among these questions, we could ask whether the presence of G4 structures does represent a major and common mechanism in gammaherpesviruses to control the production of DRiPs from nascent GMP proteins during latency? Moreover, how important is the *cis*-acting immune evasion during lymphoproliferation, a hallmark of gammaherpesvirus-associated malignancies? Indeed, a recombinant strain of AlHV-1 expressing a mutated aLANA unable to self-inhibit protein processing for presentation by MHC class I was, however, fully able to induce normal MCF. Whether this observation after AlHV-1 infection represents a general rule or just an exception is unknown. Thus, it makes no doubt that understanding the degree of involvement of GMP *cis*-acting immune evasion during gammaherpesvirus latency will be determined depending on our understanding of latency mechanisms themselves and, for instance, how distinct are silent latency in healthy individuals and latency-dependent lymphoproliferative diseases. In other words, would self-inhibition of antigen presentation by GMP represent the essential mechanism to avoid CTL recognition during gammaherpesvirus-induced lymphoproliferation? We are eager to uncover future investigations that will clarify these questions.

## Author Contributions

All authors listed have made a substantial, direct and intellectual contribution to the work, and approved it for publication.

## Conflict of Interest Statement

The authors declare that the research was conducted in the absence of any commercial or financial relationships that could be construed as a potential conflict of interest.
